# Intra-arterial Fractional Flow Reserve Measurements Provide an Objective Assessment of the Functional Significance of Peripheral Arterial Stenoses☆

**DOI:** 10.1016/j.ejvs.2023.07.035

**Published:** 2024-02

**Authors:** Mostafa A. Albayati, Ashish Patel, Bhavik Modi, Prakash Saha, Lawen Karim, Divaka Perera, Alberto Smith, Bijan Modarai, Guy's and St Thomas' Limb Salvage Research Collaborative, Lukla Biasi, Tommaso Donati, Sanjay Patel, Hany Zayed

**Affiliations:** a Academic Department of Vascular Surgery, Guy’s & St Thomas’ NHS Foundation Trust and King’s College London, United Kingdom; b Department of Cardiology, School of Cardiovascular and Metabolic Medicine and Sciences, King’s BHF Centre of Excellence, Guy’s & St Thomas’ NHS Foundation Trust and King’s College London, United Kingdom; c Guy's and St. Thomas’ NHS Foundation Trust and King's College London, United Kingdom

**Keywords:** Angioplasty, Fractional flow reserve, Haemodynamic, Peripheral arterial disease, Stenting

## Abstract

**Objective:**

Peripheral arterial stenoses (PAS) are commonly investigated with duplex ultrasound (DUS) and angiography, but these are not functional tests. Fractional flow reserve (FFR), a pressure based index, functionally assesses the ischaemic potential of coronary stenoses, but its utility in PAS is unknown. FFR in the peripheral vasculature in patients with limb ischaemia was investigated.

**Methods:**

Patients scheduled for angioplasty and or stenting of isolated iliac and superficial femoral artery stenoses were recruited. Resting trans-lesional pressure gradient (*P*_d_/*P*_a_) and FFR were measured after adenosine provoked hyperaemia using an intra-arterial 0.014 inch flow and pressure sensing wire (ComboWire XT, Philips). Prior to revascularisation, exercise ABPI (eABPI) and DUS derived peak systolic velocity ratio (PSVR) of the index lesion were determined. Calf muscle oxygenation was measured using blood oxygenation level dependent cardiovascular magnetic resonance prior to and after revascularisation.

**Results:**

Forty-one patients (32, 78%, male, mean age 65 ± 11 years) with 61 stenoses (iliac 32; femoral 29) were studied. For lesions < 80% stenosis, resting *P*_d_/*P*_a_ was not influenced by the degree of stenosis (*p* = .074); however, FFR was discriminatory, decreasing as the severity of stenosis increased (*p* = .019). An FFR of < 0.60 was associated with critical limb threatening ischaemia (area under the curve [AUC] 0.87; 95% CI 0.75 – 0.95), in this study performing better than angiographic % stenosis (0.79; 0.63 – 0.89), eABPI (0.72; 0.57 – 0.83), and PSVR (0.65; 0.51 – 0.78). FFR correlated strongly with calf oxygenation (rho, 0.76; *p* < .001). A greater increase in FFR signalled resolution of symptoms and signs (ΔFFR 0.25 ± 0.15 *vs.* 0.13 ± 0.09; *p* = .009) and a post-angioplasty and stenting FFR of > 0.74 predicted successful revascularisation (combined sensitivity and specificity of 95%; AUC 0.98; 0.91 – 1.00).

**Conclusion:**

This pilot study demonstrates that FFR can objectively measure the functional significance of PAS that compares favourably with visual and DUS based assessments. Its role as a quality control adjunct that confirms optimal vessel patency after angioplasty and or stenting also merits further investigation.

**What This Paper Adds**

Fractional flow reserve (FFR) is used to assess the haemodynamic significance of stenoses in the coronary circulation but there is a paucity of data pertaining to similar assessments for peripheral arterial lesions. FFR provides a superior functional assessment of peripheral arterial stenoses than duplex ultrasound, ankle brachial pressure index (ABPI), and angiography. A post-revascularisation FFR of ≥ 0.74 predicted successful revascularisation after percutaneous transluminal angioplasty and or stenting in the cohort of patients.

## Introduction

Peripheral arterial disease (PAD) affects 20% of individuals older than 75 years, accounting for 27 million people in Europe and North America; it can cause severe blood flow restriction leading to critical limb threatening ischaemia (CLTI).^1^ Despite the plethora of techniques for open and endovascular treatment of CLTI, a significant number of patients will eventually require amputation, suggesting a need for better diagnostic and treatment strategies.^2^ Current deficiencies in the management of these patients include the absence of reliable methodologies for objective and reproducible haemodynamic assessment of blood flow in the peripheral vasculature.

Current algorithms used to diagnose PAD and determine the ischaemic potential of peripheral arterial stenoses (PAS) are reliant on the assessment of clinical symptoms and signs, non-invasive haemodynamic testing, and angiographic assessments of lesion severity. Duplex ultrasound (DUS) and computed tomography (CT) angiography are currently the mainstay of disease severity assessment and demonstrate the patency of the main vessels. Cinefluoroscopy and digital subtraction angiography are used to confirm successful revascularisation, which is determined as an observed increase in arterial flow above an accepted visual threshold. However, this is based on arterial diameter at lesion sites, which does not objectively indicate the haemodynamic, functional status of the vessel. A surrogate for perfusion in the limb, the resting and post-exercise ankle brachial pressure index (ABPI) only crudely indicates overall flow to the foot and is limited by the presence of calcified arteries and additional disease distal to the target lesion. DUS provides some lesion level measure of haemodynamic impairment, but it is subject to interoperator variability and is also less reliable in multilevel disease.^3^ While resting intra-arterial trans-lesional pressure measurements are recommended if there is doubt about the haemodynamic significance of a lower limb lesion, these measurements do not reliably quantify the functional severity of a stenosis during exercise. Functional, haemodynamic measurements of peripheral arterial lesions could be used to support the diagnosis of limb ischaemia, indicate lesion severity, and could also represent a better on table quality control measure to confirm the adequacy of revascularisation after angioplasty and stenting. Development of such functional assessments has been identified as a key research priority in recent consensus documents, including the Global Vascular Guidelines on the Management of CLTI, and by the American Heart Association.^2^^,^^4^

In the coronary circulation, guidewire sensor technology has enabled assessment of several measures of arterial function during diagnostic coronary angiography, including trans-stenotic pressure, flow velocity, and extent of microvascular impairment. Fractional flow reserve (FFR) is an invasive pressure based index used to assess the ischaemic potential of coronary stenoses based on pressure–flow analysis during maximum hyperaemic flow.^5^^,^^6^ This objective approach assesses the functional severity of a lesion during a state of increased demand. It has translated to reduced coronary events and improved outcomes following percutaneous coronary intervention (PCI) by identifying culprit lesions that cause myocardial ischaemia and selectively targeting these for angioplasty.^5^^,^^7^ The utility of FFR for coronary revascularisation has stimulated studies that aim to apply the same principles to functional evaluation of lower limb stenoses.^8^, ^9^, ^10^

The aim was to determine (1) the utility of invasively derived FFR measurements in assessing the haemodynamic significance of peripheral arterial stenoses; and (2) the association between post-revascularisation FFR and short term clinical success.

## Materials and Methods

### Study design

This study complied with the 1975 Declaration of Helsinki with ethics approval (Guy’s & St. Thomas’ NHS Trust 10/H0804/67). Patients with short distance claudication or CLTI, scheduled for elective percutaneous transluminal angioplasty and or stenting (PTA) of isolated iliac (Trans-Atlantic Inter-Society Consensus [TASC] A – C lesions) or superficial femoral artery (SFA, TASC A – B lesions) stenoses were recruited prospectively. Patient reported symptom status and functional limitation was quantified at baseline and during follow up using a Walking Impairment Questionnaire.^11^ The decision for intervention was made by a dedicated lower limb multidisciplinary team.

Exclusions included (1) radiological evidence of more than one diseased infragenicular artery, (2) previous surgical or endovascular intervention for PAD in the target limb, (3) contraindications to magnetic resonance imaging scanning, and (4) patients unable to consent.

Pre-procedural investigations included resting ABPI (rABPI) and exercise ABPI (eABPI), DUS, CT angiography, and assessment of calf oxygenation by blood oxygenation level dependent (BOLD) cardiovascular magnetic resonance (CMR). All procedures were performed under local anaesthesia in a hybrid operating suite using a Philips AlluraXper FD20 system (Philips Healthcare, Eindhoven, The Netherlands). Intravascular Doppler derived flow reserve and pressure derived FFR were obtained during PTA, followed by repeat BOLD CMR scanning after revascularisation. Clinical status was assessed during follow up and target vessel patency determined by DUS.

### Non-invasive assessments

#### Ankle brachial pressure index and peak systolic velocity ratio

Pre-procedural rABPI and eABPI measurements of the target limb and pre-procedural colour DUS of the target lesion were obtained. For the purposes of the present study, a peak systolic velocity ratio (PSVR) of ≥ 2.4 (equivalent to ≥ 50% lumen diameter narrowing) was defined as a significant stenosis on DUS and target vessel patency, assessed by DUS four months after intervention, was defined as a PSVR ≤ 2.4 in the treated segment.

#### Computed tomography angiography

Pre-procedural CT angiography was performed from the diaphragm to the foot arches with image slice thickness of 1 mm, using a 64 slice Philips Brilliance iCT scanner (Philips Healthcare, Best, The Netherlands). A single observer quantified the percentage diameter stenosis (% DS) manually using cross sectional images at the most severe site of narrowing and in the proximal non-diseased reference arterial segment. From these two images, % DS was calculated as (1 – *L*/*R*) × 100, where *L* is minimum lesion diameter and *R* is diameter at the proximal reference site.

#### Blood oxygenation level dependent cardiovascular magnetic resonance

Calf muscle perfusion during hyperaemia was measured using BOLD-CMR as described previously.^12^ BOLD-CMR images of the calves were obtained on the same day immediately prior to intervention and repeated after revascularisation, using a 3-T Philips Achieva scanner (Philips Healthcare, Best, The Netherlands) with a multi-echo single shot gradient recalled echo (GRE) sequence. All patients refrained from caffeine and exercise 12 hours before imaging and rested supine for five minutes, in order to standardise scans. Reactive hyperaemia, provoked by cuff induced arterial occlusion followed by rapid cuff deflation, was used to elicit T2∗ changes. Regions of interest (ROIs) were drawn around the five muscle groups (anterior, lateral, soleus, gastrocnemius, and deep posterior) in each leg, using the GRE-BOLD T2∗ maps with the TSE images providing a visual guide. Bespoke MATLAB (MathWorks, Natick, MA, USA) routines were used to automatically generate time course curves for individual ROIs and analyse curve parameters. Images were analysed by the same blinded user.

### Invasive pressure and Doppler flow measurements

Intravascular haemodynamic measurements were obtained using a 0.014 inch (0.36 mm) dual sensor Doppler flow and pressure derived guidewire (ComboWire XT, Philips Healthcare, Eindhoven, The Netherlands) and workstation (ComboMap, Philips Healthcare, Eindhoven, The Netherlands). This workstation was not viewable to the operator to minimise the influence of recorded haemodynamic data on procedural decision making.

**Figure 1:**
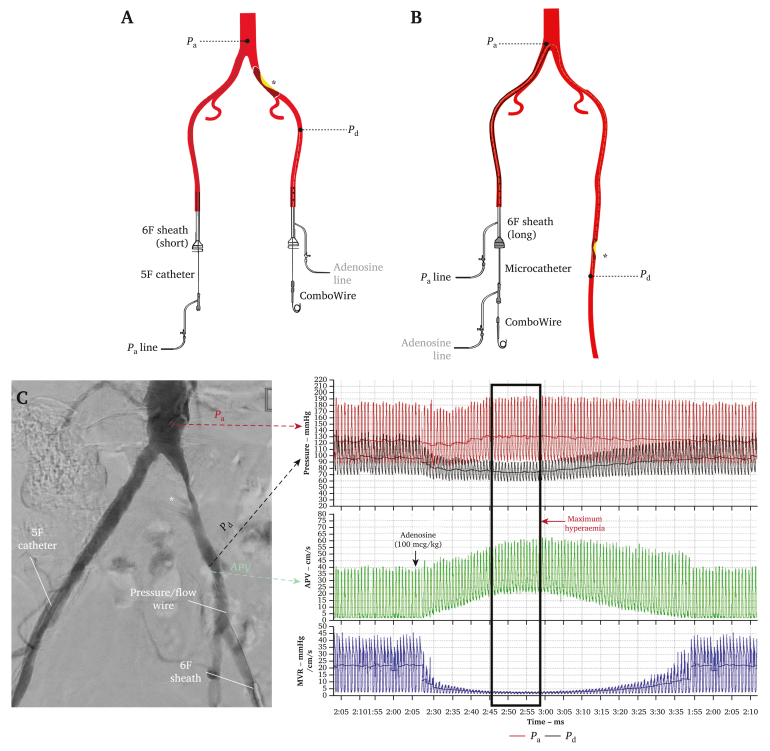
Procedural steps for measuring resting pressure (*P*_d_/*P*_a_) and fractional flow reserve (FFR). (A) For iliac artery stenoses, resting aortic pressure, *P*_a_, is obtained via a 5 French catheter placed in the distal aorta using contralateral femoral access. Pressure distal to the index lesion (asterisk), *P*_d_, is acquired concurrently using a ComboWire inserted via the ipsilateral femoral sheath. Adenosine is administered distal to the index lesion via the ipsilateral femoral sheath. FFR is defined as the lowest *P*_d_/*P*_a_ ratio following administration of adenosine. (B) For superficial femoral artery stenoses, single femoral access from the contralateral limb was obtained and a long 6 French sheath to measure *P*_a_. The lesion was crossed with a ComboWire and microcatheter, which was used to administer adenosine then immediately fully retracted. The ComboWire is kept in place to allow measurement of *P*_d_ distal to the lesion. FFR is defined as the lowest *P*_d_/*P*_a_ ratio following administration of adenosine. (C) Representative digital subtraction angiogram of a left common iliac artery stenosis demonstrating bilateral access with sheath, catheter, and ComboWire positioning. Subsequent *P*_d_ (black), *P*_a_ (red) and average peak velocity (APV) (green) waveforms at rest and during adenosine mediated hyperaemia were acquired. MR = microvascular resistance.

Iliac stenoses were approached via bilateral femoral access and SFA stenoses were treated using a contralateral, up and over approach according to standard institutional protocols (Fig. 1). Prior to insertion, the ComboWire was calibrated to zero then equalised to the catheter tip position in the distal aorta following insertion. Simultaneous recording of distal aortic pressure (*P*_a_), pressure distal to the target stenosis (*P*_d_), and average peak velocity (APV) were obtained. At the end of each recording, the ComboWire sensor was returned to the catheter tip to assess pressure drift. If pressure drift was identified (> 2 mmHg) measurements were repeated or corrected for during offline analysis.

#### Measurement of pressure index and fractional flow reserve

Hyperaemia was induced using 100 μg/kg adenosine administered as an intra-arterial bolus distal to the index lesion, followed by 5 mL saline flush. This dose was selected based on dose response studies demonstrating its maximum hyperaemic effect when administered intra-arterially in coronary and peripheral vascular beds.^13^^,^^14^ The resting pressure index (*P*_d_/*P*_a_) was calculated as the ratio of baseline mean *P*_d_ to mean P_a_. FFR was defined as the lowest *P*_d_/*P*_a_ ratio following administration of adenosine, averaged over five cardiac cycles.^15^ Haemodynamic measurements were obtained from contralateral non-diseased iliac arteries to determine reference values.

Data were processed offline for accurate waveform analysis of all baseline and hyperaemic tracings. Tracings with significant artefact, dampened waveforms, or non-correctable sensor drift were excluded.

### Statistical analysis

The association between variables and disease severity was assessed using receiver operating characteristic (ROC) curve analysis. Optimal FFR cut off values for (1) determining the relationship between intermittent claudication and CLTI, and (2) predicting re-intervention were defined with Youden’s index (*J*), calculated as [(sensitivity + specificity) – 1], where the sum of sensitivity and specificity are maximised.

Correlations were determined using Pearson, Spearman, or Kruskal–Wallis tests and differences in variables across progressive % DS strata and walking distance were assessed using one way analysis of variance. Analyses were performed using SPSS version 26 (IBM Corp., Armonk, NY) and Prism 8.4 (GraphPad Software Inc., La Jolla, CA) on a per lesion basis and *p* values < .05 were considered statistically significant.

## Results

Sixty-one stenoses (iliac, *n* = 32; femoral, *n* = 29) in 41 patients (10 with bilateral disease) were evaluated (Table 1). Fifty-two lesions with a mean CT derived % DS of 67 ± 17%, underwent PTA. The clinical indication for treatment was short distance claudication in 58% (*n* = 30) and CLTI in 42% (*n* = 22), and the mean duration of symptoms and signs was 26 ± 15 months. The CLTI cohort included seven (32%) with chronic ulcers. The remaining nine iliac stenoses studied were < 30 % DS, identified from the pre-procedural CT angiogram. These were not intended to be treated, but invasive haemodynamic analysis of these subthreshold stenoses was carried out during treatment of the unrelated target lesion. Measurements were also obtained in 16 non-diseased iliac arteries in the contralateral limb to provide reference haemodynamic values to understand further the hyperaemic pressure gradient–flow velocity relationship in both healthy and diseased vessels, and to aid in assessing the diagnostic performance of FFR. There were no complications from the use of the dual sensor guidewire or adenosine administration. All lesions were treated with balloon angioplasty and stenting. Angiographic success, defined as < 30% residual stenosis after PTA, was achieved in all cases.

**Table 1:** Patient demographics, disease severity, and stenosis characteristics of limbs undergoing percutaneous transluminal angioplasty (PTA)

Characteristics	Patients (*n* = 41)
Age – y	65 ± 11
Male	32 (78)
Hypertension	30 (73)
Hypercholesterolaemia	28 (68)
Diabetes mellitus	13 (32)
Smoking	31 (76)
IHD	15 (37)
Aspirin	26 (63)
Clopidogrel	15 (37)
Statin	30 (73)
Antihypertensive	33 (80)
Antihyperglycaemic	10 (24)
*Rutherford classification of treated limbs*∗	
Rutherford 3	30 (58)
Rutherford 4	15 (29)
Rutherford 5	7 (13)
*TASC classification*∗	
*Aorto-iliac*	
Type A	7 (13)
Type B	9 (17)
Type C	8 (16)
*Femoropopliteal*	
Type A	15 (29)
Type B	13 (25)

Data are presented as mean ± standard deviation or *n* (%). IHD = ischaemic heart disease; TASC = Trans-Atlantic Intersociety Consensus.

∗ *n* = 52

### Relationship between anatomical stenosis severity and pressure–flow measurements

**Figure 2:**
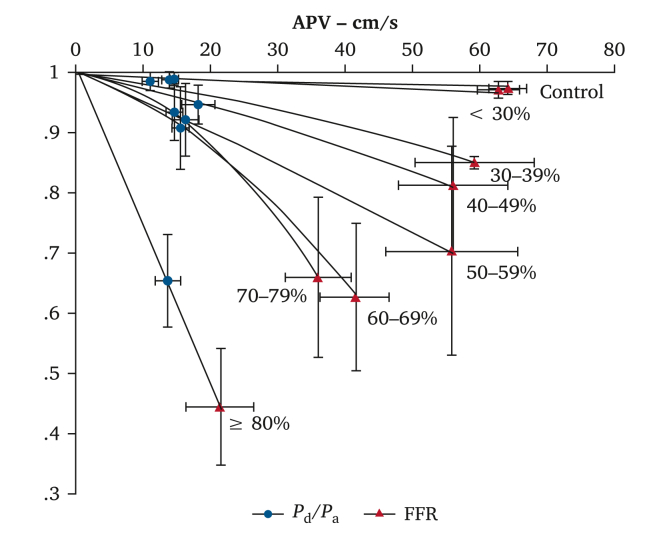
Relationship between resting and hyperaemic pressure indices and lesional diameter stenosis measured angiographically. In non-stenosed arteries and those with minimal stenoses (< 30% diameter stenosis), *P*_d_/*P*_a_ (distal lesion pressure/distal aortic pressure) (black circles) and fractional flow reserve (FFR) (red triangles) are comparable. Lesions in the 30 – 79% diameter stenosis range were not well discriminated using resting *P*_d_/*P*_a_ index and required FFR measurement after provocation of hyperaemia by adenosine to unmask the true extent of haemodynamic impairment. Lesions over 80% diameter stenosis demonstrated lower *P*_d_/*P*_a_ values and could be haemodynamically discriminated from lesions less than 80% diameter stenosis using resting *P*_d_/*P*_a_ measurements alone. Curves fitted using second order polynomial, and bars represent standard error of mean. APV = average peak velocity.

Mean *P*_d_/*P*_a_ and FFR values corresponding to the 16 non-diseased iliac vessels were 0.98 ± 0.01 and 0.96 ± 0.03, respectively, demonstrating that no significant drop in pressure occurred along a normal major lower limb artery. ‬In‬ lesions < 80 % DS, ‬‬‬resting trans-lesional pressure measurements (*P*_d_/*P*_a_) were not influenced by the degree of stenosis (*p* = .074); however, FFR measurements were discriminatory, decreasing with incremental % DS severity (*p* = .019, Fig. 2). Both *P*_d_/*P*_a_ and FFR in lesions ≥ 80 % DS were significantly lower than in 30 – 79 % DS both at rest (*p* < .001) and during hyperaemia (*p* = .002). ‬‬‬‬‬‬‬‬‬‬‬‬‬‬‬‬‬‬‬‬‬‬‬‬‬‬‬‬‬

#### Association between intra-arterial pressure–flow measurements and clinical disease severity

**Figure 3:**
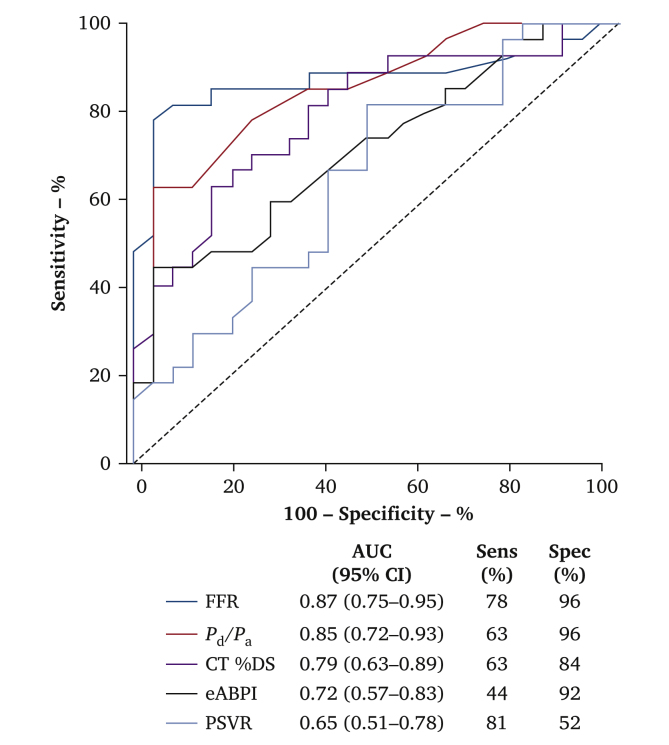
Association between standard of care assessments and intra-arterial pressure–flow measurements with critical limb threatening ischaemia. Receiver operator characteristic curve analysis with corresponding area under the curve (AUC), 95% confidence interval (CI), and sensitivity (Sens) and specificity (Spec) values. Fractional flow reserve (FFR) demonstrates the greatest AUC for association with critical limb threatening ischaemia in this cohort of patients. *P*_a_ = distal aortic pressure; *P*_d_ = distal lesion pressure; CT % DS = computed tomography percentage diameter stenosis; eABPI = exercise ankle brachial pressure index; PSVR = peak systolic velocity ratio.

The association between imaging findings, non-invasive and invasive haemodynamic measurements, and presence of CLTI or intermittent claudication is shown in Figure 3. A lower FFR (area under the receiver operating characteristic curve [AUC] 0.87; 95% CI 0.75 – 0.95) was associated with CLTI in the cohort studied, as was a lower *P*_d_/*P*_a_ (AUC 0.85; 95% CI 0.72 – 0.93). The degree of lesional stenosis measured on CT (AUC 0.79; 0.63 – 0.89) by eABPI (AUC 0.72; 0.57 – 0.83) and PSVR (AUC 0.65; 0.51 – 0.78) had a weaker association with CLTI.

### Correlation of fractional flow reserve with limb tissue oxygenation

**Figure 4:**
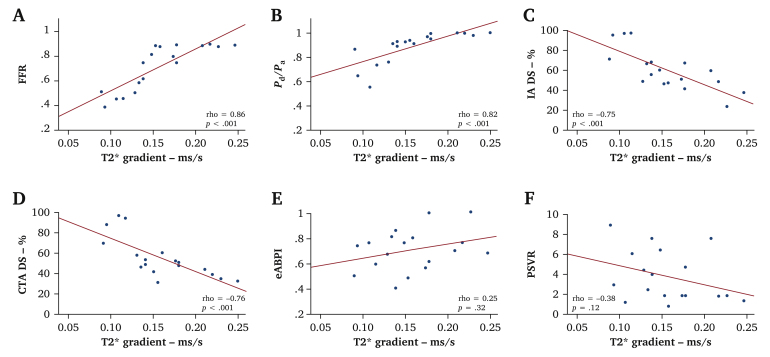
Scatterplot of blood oxygenation level dependent cardiovascular magnetic resonance derived limb perfusion and corresponding angiographic metrics, and non-invasive and invasive haemodynamic indices. Relationship between limb tissue oxygenation (T2∗ gradient) and (A) fractional flow reserve, (B) *P*_d_/*P*_a_ (distal lesion pressure/distal aortic pressure), (C) computed tomography percentage diameter stenosis (CT % DS), (D) invasive angiography percentage diameter stenosis (IA % DS), (E) peak systolic velocity ratio (PSVR), and (F) exercise ankle brachial pressure index (eABPI).

Calf oxygenation was assessed by BOLD-CMR prior to PTA in 14 of the 52 patients recruited to the study (18 ischaemic limbs and 10 healthy contralateral control limbs). Pre-PTA T2∗ gradient, indicating calf muscle oxygenation status, correlated strongly with the FFR index (rho, 0.76; *p* < .001), and moderately with *P*_d_/*P*_a_ (0.54; *p* = .022, Fig. 4). There was no correlation between calf oxygenation and angiographic % DS (invasive –0.43, *p* = .077; CT –0.38, *p* = .12) or with PSVR (–0.06; *p* = .80), and eABPI (0.03; *p* = .91). BOLD-CMR was repeated after revascularisation in 12 limbs. Post-revascularisation FFR correlated strongly with the corresponding T2∗ Grad (rho, 0.79; *p* < .001) after revascularisation. There was no correlation between post-PTA T2∗ and *P*_d_/*P*_a_ (*p* = .18), ΔPSVR (*p* = .25), and eABPI (*p* = .93).

### Post-revascularisation fractional flow reserve and short term clinical outcomes

**Figure 5:**
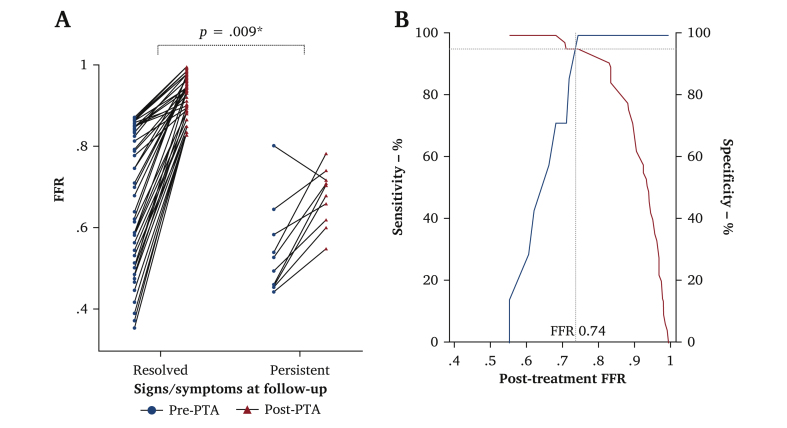
Change in fractional flow reserve (FFR) values after angioplasty and stenting of lesions and association between post-intervention FFR and treatment failure. (A) Per lesion change in FFR following angioplasty and stenting of index lesion, grouped according to patients with clinical improvement and those with persistent signs and symptoms at follow up. (B) Post-percutaneous transluminal angioplasty (PTA) FFR cut off value corresponding to the maximum Youden index for predicting revascularisation. ∗Comparison of mean ΔFFR (pre- and post-PTA) between the two groups of resolved and persistent symptoms.

The mean *P*_d_/*P*_a_ and FFR indices prior to revascularisation in the 52 treated stenoses were 0.85 ± 0.12 and 0.65 ± 0.17 and improved to 0.92 ± 0.08 and 0.88 ± 0.09 (*p* < .001 for both) after PTA, respectively. After a median follow up of four months (interquartile range [IQR] 3, 5), successful resolution of CLTI or intermittent claudication was confirmed in 42 of the 52 target limbs. Persistent claudication, rest pain, and non-healing wounds accounted for failed revascularisation in the remaining 10 limbs (Table 2). There was a greater increase in post-revascularisation FFR in patients with successful revascularisation than in those who had a failed intervention (ΔFFR 0.25 ± 0.15 *vs.* 0.13 ± 0.09, respectively, *p* = .009, Fig. 5). A post-PTA FFR index of > 0.74 predicted successful revascularisation (AUC 0.98; 0.91 – 1.00) with a maximum combined sensitivity and specificity of 95%.

**Table 2:** Lesion characteristics, haemodynamic indices and clinical outcome in patients who did not improve after angioplasty and stenting of index lesion

Case	Clinical signs and symptoms	Lesion characteristics	Pre-PTA FFR	Post-PTA FFR	Clinical outcome	Re-intervention
1	Rest pain	CIA (63 % DS)	0.49	0.62	Persistent pain	Yes
2	Foot ulcer	SFA (92 % DS)	0.45	0.68	Non-healing ulcer	Yes
3	SDC	CIA (63 % DS)	0.54	0.78	Persistent pain	No
4	SDC	EIA (61 % DS)	0.65	0.74	Persistent symptoms	Yes
5	SDC	EIA (71 % DS)	0.53	0.71	Persistent symptoms	Yes
6	SDC	EIA (62 % DS)	0.80	0.72	Persistent symptoms	No
7	Rest pain	SFA (81 % DS)	0.58	0.66	Persistent pain	Yes
8	Rest pain	CIA (72 % DS)	0.46	0.71	Persistent pain	No
9	Foot ulcer	SFA (94 % DS)	0.46	0.60	Non-healing ulcer	Yes
10	Foot ulcer	SFA 97 % DS)	0.44	0.55	Non-healing ulcer	Yes

% DS = percentage diameter stenosis; PTA = percutaneous transluminal angioplasty; FFR **=** fractional flow reserve; SDC = short distance claudication; CIA = common iliac artery; EIA = external iliac artery; SFA = superficial femoral artery.

## Discussion

The present study demonstrates that haemodynamic assessments using FFR may provide a better indication of the functional significance of peripheral arterial stenoses than DUS and angiography. Pre-treatment FFR demonstrated superiority in disclosing the haemodynamic significance of stenoses that were less than 80% of the vessel diameter and correlated strongly with calf oxygenation. Post-procedural improvements in FFR indicated the likelihood of successful revascularisation and clinical resolution of ischaemia.

CT and intra-arterial angiography are currently used to inform the anatomical extent of PAD and assess lesions for intervention. However, the degree of lumen diameter narrowing may not reliably indicate the haemodynamic significance of a stenosis particularly for those in the intermediate range. Visual assessment of the degree of stenosis is also prone to significant interobserver variation.^16^ Although DUS provides a non-invasive measure of lesion specific haemodynamic impairment, this is performed during resting conditions, which may partly explain its weak correlation with FFR based functional lesion assessment as well as with clinical symptoms and signs. In practice, if appropriate clinical signs and symptoms of limb ischaemia are supported by DUS or angiography findings then revascularisation is likely to be carried out, but in some patients these clinical findings and or arterial assessments can be equivocal and raise confusion about the clinical importance of a given peripheral arterial lesion. This may, in part, account for the large regional variations in treatment practices and clinical outcomes for patients with PAD.^17^

In the coronary circulation a lesion associated with a FFR of < 0.8 is deemed haemodynamically significant, with three clinical trials demonstrating that FFR guided intervention leads to reduced major adverse cardiac events as well as lower treatment costs due to reduced stent use compared with solely angiographically guided revascularisation.^18^, ^19^, ^20^, ^21^

Resting trans-lesional pressures, rather than FFR measurements, are increasingly used in the coronary circulation to simplify the workflow and avoid the need for provoking hyperaemia.^22^ The present study demonstrates, however, that in the peripheral vasculature adenosine induced vasodilation is needed to unmask the haemodynamic significance of some lesions, suggesting the FFR may better inform on table revascularisation decisions in stenoses of equivocal anatomical severity. In patients with intermittent claudication, symptoms occur when blood supply is unable to meet the muscle perfusion demand during an exercise induced hyperaemic state. This probably explains why FFR correlated better with ischaemic burden when compared with the resting *P*_d_/*P*_a_ index and is, therefore, also superior to trans-lesional resting pressure gradients with a catheter that has historically been used intra-operatively to decipher the significance of peripheral arterial stenoses.

It was demonstrated that the lower limb vasculature has an autoregulatory process maintaining stable resting blood flow similar to that seen in the coronary vascular bed.^23^ During resting flow, *P*_d_/*P*_a_ remains fairly constant across the spectrum of stenoses below 80% luminal narrowing and masks the haemodynamic significance of these lesions. This preservation of trans-stenotic pressure is mediated by a compensatory reduction in microvascular resistance in response to increasing stenosis resistance. The peripheral vascular bed responds sufficiently to adenosine mediated vasodilation, thereby lowering microvascular resistance and increasing blood flow within the vessel. This ability to minimise the autoregulatory capacity of the microvascular resistance supports the clinical use of hyperaemic pressure measurement to determine the functional significance of lower limb stenoses. The present study highlights that a functional deficit (confirmed with a low FFR value) may be present despite a moderate lesion assessed by % DS on CT, reflecting impairment of the distal microvasculature rather than main vessel disease. This has been found with coronary stenoses, where FFR has been used more extensively.

BOLD-CMR can be used to measure calf muscle oxygenation in the lower limb by quantifying the T2∗ signal changes.^12^^,^^24^ In the present study, FFR correlated with the hyperaemic T2∗ signal in ischaemic limbs both before and after revascularisation. By contrast, there was poor concordance between T2∗ signal and lesional stenosis measured by CT angiography, as well as with the stress induced of exercise ABPI. The findings also suggest that FFR may better predict successful restoration of tissue oxygenation after limb revascularisation than existing clinical imaging tests.

While FFR has primarily been used to determine the need for PCI and direct coronary artery revascularisation, much less is known about its prognostic value after revascularisation. Approximately 30 – 40% of patients who undergo above knee stent implantation for PAD will develop in stent re-stenosis within two years of intervention.^25^ Intra-procedural success of PTA is currently determined by invasive angiography, assessing only the morphology of the treated segment. In coronary lesions, a persistently low FFR following PCI is associated with worse clinical outcomes, though an optimal threshold for post-PCI FFR has not been determined.^26^ Treatment failure at follow up was found to be associated with a smaller improvement in FFR after PTA. Potential explanations for a low FFR after revascularisation include suboptimal stent placement or sizing, underexpansion, residual stenosis due to dissection or plaque, or further distal atherosclerotic disease impacting accurate FFR measurement. In addition, patients with persistent distal microvascular dysfunction would have little change in FFR after treatment and their clinical symptoms and signs would fail to resolve. The analysis of post-revascularisation FFR in patients with persistent symptoms and signs at follow up showed that a post-PTA FFR of > 0.74 indicates successful revascularisation with high sensitivity and specificity. This highlights a further potential application for FFR to confirm adequate treatment, as well as identifying the need for additional endovascular procedures to optimise outcomes. It would also identify patients who may benefit from closer follow up to mitigate against re-stenosis or re-intervention.

One limitation of the model of FFR measurement outlined in the present study is that it requires invasive arterial access for lesions that may subsequently prove to be haemodynamically insignificant. CT derived calculation of FFR using computational fluid dynamics (CFD) in patients with stable coronary disease has been proposed to circumvent the need for invasive FFR measurements.^27^ This imaging modality could be translated to the lower limb vasculature to non-invasively interrogate functional stenosis severity and avoid unnecessary invasive angiography. However, this technique relies on high quality, detailed reconstructed CT angiography images of the lower limb vasculature and requires significant computational power to run CFD modelling simulations in a timely manner that would then need validation with invasively derived haemodynamic measurements.^28^

### Study limitations

In this proof of concept study, the inclusion criteria were deliberately limited to patients with a single above knee arterial lesion to minimise confounders and facilitate development of a repeatable and reliable technique, including understanding the role of adenosine in stimulating a haemodynamic stress response. The CLTI population represented in the present study is, therefore, highly selected. Further studies in patients with multilevel disease, including patients with extensive co-existing below knee lesions, are warranted before the results can be applied to a wider group of patients. Although this study was designed and performed in a prospective manner, the sample size was relatively small. Further studies with large sample sizes are warranted to substantiate the finding that post-intervention FFR predicts re-stenosis. The presence of compensatory collateral vessels, which could influence the total blood flow, and therefore FFR index, was not studied. Finally, the operator was blinded to the FFR indices and waveform analyses were performed offline, but using an independent core laboratory would be the preferred mode of analysis.

### Conclusion

This pilot study demonstrates that fractional flow reserve can objectively measure the functional significance of peripheral arterial stenoses and may prove useful as an adjunct that personalises treatment algorithms, including as a quality control tool to ensure optimal vessel patency after revascularisation, reducing the need for re-intervention and ultimately promoting limb salvage.

## Conflict of Interest

Bijan Modarai: Consulting, speakers fees and research grants from Cook, Philips, and Cydar Medical.

## Funding

Dr Albayati was funded by the 10.13039/100000002National Institutes of Health Research Biomedical Research Centre at Guy’s and St Thomas’ NHS Foundation Trust and King’s College London and British Heart Foundation Clinical Research Training Fellowships (FS/16/50/32337). Professor Modarai is funded by a British Heart Foundation Senior Clinical Research Fellowship (FS/17/24/32596).
