# Supplementary material for: Intra-arterial Fractional Flow Reserve Measurements Provide an Objective Assessment of the Functional Significance of Peripheral Arterial Stenoses
Source: Eur J Vasc Endovasc Surg. 2024 Feb;67(2):332–40. doi: 10.1016/j.ejvs.2023.07.035 (PMC10917690; doi:10.1016/j.ejvs.2023.07.035)
Supplement: Supplementary Material [file mmc1.pdf]

Appendix 1

Guy's and St Thomas' Limb Salvage Research Collaborative

Lukla Biasi, Tommaso Donati, Sanjay Patel and Hany Zayed

Affiliation: Guy's and St. Thomas' NHS Foundation Trust and King's College London
